# Small interfering RNA against CD86 during allergen challenge blocks experimental allergic asthma

**DOI:** 10.1186/s12931-014-0132-z

**Published:** 2014-10-27

**Authors:** Yukari Asai-Tajiri, Koichiro Matsumoto, Satoru Fukuyama, Keiko Kan-o, Takako Nakano, Ken Tonai, Tatsukuni Ohno, Miyuki Azuma, Hiromasa Inoue, Yoichi Nakanishi

**Affiliations:** Research Institute for Diseases of the Chest, Graduate School of Medical Sciences, Kyushu University, Fukuoka, Japan; Department of Molecular Immunology, Graduate School, Tokyo Medical and Dental University, Tokyo, Japan; Department of Pulmonary Medicine, Graduate School of Medical and Dental Sciences, Kagoshima University, Kagoshima, Japan

## Abstract

**Background:**

CD86-CD28 interaction has been suggested as the principal costimulatory pathway for the activation and differentiation of naïve T cells in allergic inflammation. However, it remains uncertain whether this pathway also has an essential role in the effector phase. We sought to determine the contribution of CD86 on dendritic cells in the reactivation of allergen-specific Th2 cells.

**Methods:**

We investigated the effects of the downregulation of CD86 by short interfering RNAs (siRNAs) on Th2 cytokine production in the effector phase *in vitro* and on asthma phenotypes in ovalbumin (OVA)-sensitized and -challenged mice.

**Results:**

Treatment of bone marrow-derived dendritic cells (BMDCs) with CD86 siRNA attenuated LPS-induced upregulation of CD86. CD86 siRNA treatment impaired BMDCs’ ability to activate OVA-specific Th2 cells. Intratracheal administration of CD86 siRNA during OVA challenge downregulated CD86 expression in the airway mucosa. CD86 siRNA treatment ameliorated OVA-induced airway eosinophilia, airway hyperresponsiveness, and the elevations of OVA-specific IgE in the sera and IL-5, IL-13, and CCL17 in the bronchoalveolar lavage fluid, but not the goblet cell hyperplasia.

**Conclusion:**

These results suggest that local administration of CD86 siRNA during the effector phase ameliorates lines of asthma phenotypes. Targeting airway dendritic cells with siRNA suppresses airway inflammation and hyperresponsiveness in an experimental model of allergic asthma.

## Background

Interaction of antigen-presenting cells (APCs) and T cells is crucial in both the initiation and challenge phases of allergic asthma and, therefore, has possibility as a target of anti-asthmatic drugs. Optimal T cell activation and differentiation require not only interaction between the T cell receptor (TCR) and antigen-MHC complexes but also interaction between costimulatory ligands on APCs and their putative receptors on T cells. One of the best-characterized costimulatory molecules is CD28, which binds to two costimulatory ligands, B7-1 (CD80) and B7-2 (CD86), on APCs. CD28 is constitutively expressed on both CD4^+^ and CD8^+^T cells. By contrast, CD80/86 expression on dendritic cells and B cells is upregulated after antigen pulse in the process of maturating into APCs. Despite sharing the same receptor, CD80 and CD86 appear to mediate different mechanisms. CD80 can be more potent than CD86 in inducing antitumor responses, while CD86 preferentially induces Th2-driven allergic responses [1,2]. It is generally accepted that during APC/T cell interaction, the B7-CD28 pathway is indispensable for the activation and differentiation of naïve T cells. However, it remains controversial whether this pathway has a pivotal role in the reactivation of primed T cells in the effector phase.

It is well known that the dendritic cell (DC) is the most powerful APC for inducing allergic immune responses *in vivo*. The DC network beneath the epithelium of the conducting airways is ideally positioned to perform a surveillance role for inhaled antigens (Ag). By depleting DCs before the inhaled Ag challenge, all the salient features of asthma were diminished, and the effector cytokine secretion was profoundly reduced [3]. Moreover, recent studies suggest that the expression of CD86, but not CD80, on airway DCs is upregulated during the effector phase. Ongoing allergic inflammation induces a specific shift in airway DCs from a CD86-low to a CD86-high phenotype in periphery [4]. Given that lung DCs maturate and upregulate the expression of CD86 following the allergen challenge [5], it is important to know whether the upregulation of CD86 has a role in the development of asthmatic responses.

RNA interference is an endogenous cellular mechanism in which short interfering RNAs (siRNAs) elicit the sequence-specific degradation of a complementary mRNA target. Today, siRNA-mediated gene silencing has become more powerful, more specific, and much less toxic than low-molecular-weight chemical inhibitors or blocking mAbs for laboratory investigations, particularly *in vitro*. It is also characterized by a low-cost and transient effect. Several studies have demonstrated the therapeutic effects of synthetic siRNA in allergen-induced asthma models [6-8]. These studies targeted STAT6, TRAIL, and PAI-1, and the siRNAs were delivered by intratracheal administration. Although low transfection efficacy might be a potential hurdle in RNA interference, it is expected that DCs can be good targets because of their location and their prominent particle uptake ability, including the uptake of short-based nucleotides. Here, we examined the effects of the downregulation of CD86 by intratracheal administration of siRNA on Th2 cytokine production in the effector phase *in vitro* and on asthma phenotypes *in vivo*.

## Methods

### Mice

BALB/c mice were purchased from SLC (Hamamatsu, Japan). OVA-specific TCR-expressing DO11.10 transgenic (Tg) mice were provided by Dr. K. Murphy (Washington University, St. Louis, MO). All experimental procedures were approved by the animal research ethics committee of Kyushu University (reference number: A23-048-1).

### siRNA preparation

The sequences of the CD86 siRNA were previously determined by our colleague [9]. siRNAs were synthesized using Qiagen 2-for-silencing siRNA duplexes (Qiagen, Valencia, CA). CD86 siRNA has the sequence 5′-CGUUGUGUGUGUUCUGGAAdTdT-3′ (sense) and 5′-UUCCAGAACACACACAACGdTdT (antisense). As a control, we used single non-targeting siRNAs.

### Transfection

Bone marrow-derived DCs (BMDCs) were obtained as previously described [10], washed with a serum-free medium, and cultured in a 24-well tissue culture plate with 2 × 10^5^ cells/500 μl. Transfection reagent (Gene Silencer®, Genlantis) and siRNA were added to the wells. After 4 hours, 500 μl medium with 20% FBS was added. After 24 hours, lipopolysaccharide (LPS) (1 μg/ml; Sigma, St. Louis, MO, USA) and peptide (p) OVA (C-terminal 323–339 epitope) (5 μg/ml; Bachem, Hauptstrasse, Switzerland) were added for 18 hours.

### Flow cytometry

The cells were stained with FITC-conjugated anti-CD11c mAb and biotinylated anti-CD86 mAb followed by phycoerythrin (PE)-conjugated streptavidin (BD Biosciences). FITC-conjugated mouse IgG and PE-conjugated IgG were used as isotype controls. Flow cytometry was performed on a FACSCalibur flow cytometer equipped with CELLQuest software (BD Biosciences). Data was assessed by mean fluorescent intensity (MFI) or histogram.

### Coculture with Th2 cells

CD4^+^T cells were isolated from the spleens of naïve DO11.10 mice using magnetic separation (MACS; Miltenyi Biotec, Bergisch Gladbach, Germany). OVA-specific Th2 cells were induced as previously described [11] and cultured at a final concentration of 1 × 10^6^ cells/well/500 μl with siRNA-transfected BMDCs (at 2 × 10^5^ cells/well/500 μl). The culture supernatants were collected 48 hours later.

### Asthma model

BALB/c mice were sensitized with an intraperitoneal injection of 10 μg OVA (GradeV, Sigma-Aldrich, St. Louis, MO) with 0.3 mg of Al(OH)_3_ (SERVA Electrophoresis) on days 1 and 14. On days 26–28, animals were anesthetized and treated with intratracheal CD86 siRNA (12.5 μg siRNA in 50 μl saline). One hour after each treatment, mice were challenged with aerosolized 1% OVA for 30 minutes. On day 30, mice were assessed for airway hyperresponsiveness (AHR) followed by blood sampling and bronchoalveolar lavage (BAL) as previously described [12].

### Histology

Fresh-frozen sections were prepared with Kawamoto’s method for maintaining a normal structure [13]. For fluorescence microscopy, cryosections were stained with FITC-conjugated anti-CD11c mAb(clone HL3) or FITC-conjugated anti-CD86 mAb(clone GL1) (BD Biosciences). Images were acquired using an Olympus (Melville, NY) BX61 upright microscope. To assess the localization of siRNA, sensitized mice were intratracheally administrated Texas Red-labeled siRNA (40 μM/50 μl/animal, siGLO®) on day 26, followed by the OVA challenge. Twelve hours after the challenge, cryosections of the tracheas and lungs were prepared, stained with FITC-labeled anti-CD11c mAb, and viewed on an A1Rsi confocal laser microscope (Nikon, Tokyo, Japan). Micrographs were quantitatively analyzed for the presence of anti-CD86 signals with image analysis software. For each naïve or control siRNA-treated or CD86siRNA-treated sample, 3-4 sections were quantified. To evaluate only CD86-expressing cells, we selected an area between the airway epithelium and the lamina propria mucosae, and counted only dot signals per unit area. Goblet cell hyperplasia was assessed by staining with Alcian blue/periodic acid-Schiff (AB/PAS) as described previously [14].

### Determination of MUC5AC mRNA

Total RNA was isolated and quantitative real-time PCR was conducted for the detection of MUC5AC as described previously [11].

### Measurement of cytokines, chemokine and OVA-specific IgE

Cytokines, chemokine and OVA-specific IgE were quantified using ELISA kits (Invitrogen Corporation or R&D Systems, Shibayagi for IgE).

### Statistical analysis

Values are expressed as the mean (±SEM). Differences among groups were analyzed using an ANOVA with a Bonferroni analysis. Nonparametric data were analyzed using the Mann-Whitney U test. A *P* value of < .05 was considered statistically significant.

## Results

### siRNA downregulates CD86 expression on BMDCs

**Figure 1:**
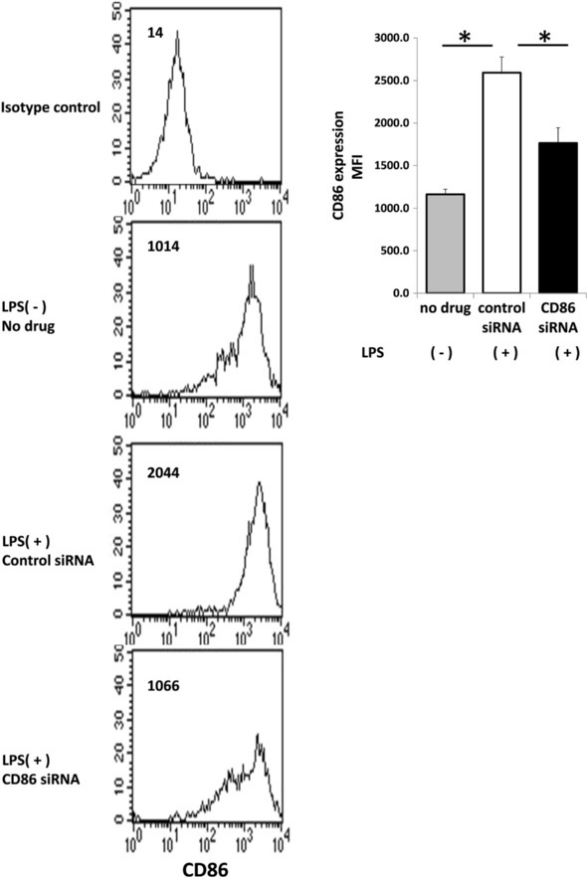
**Silencing CD86 expression by transfection with CD86 small interfering RNA (siRNA) on BMDCs.** BMDCs were cultured in the presence of GM-CSF for 7 days. The DCs were transfected with 100nM CD86 siRNA and non-specific (control) siRNA by GeneSilencer®. Twenty-four hours after gene silencing, DCs were activated with LPS for 18 hours. Cells were stained with FITC-conjugated anti-CD11c mAb and biotinylated anti-CD86 mAb followed by phycoerithrin (PE)-conjugated streptavidin. CD86 expression on CD11c-positive cells was assessed by flow cytometry. Histograms represent the number of cells at various fluorescent intensities. MFI data was expressed as the mean ± SEM of 4 mice. * indicates statistically significant (*P* <0.05) difference between the indicated two groups.

We first investigated the efficacy of CD86 siRNA in BMDCs. Immature BMDCs showed moderate expression of CD86, which was upregulated by maturation-inducing stimulation with LPS and pOVA for 18 hrs. We compared the expression level of CD86 between non-targeting control siRNA-treated and –untreated BMDCs that were stimulated with LPS and pOVA. There was no significant difference in CD86 expression (control siRNA-treated BMDCs, MFI 2593 ± 193; untreated BMDCs, MFI 3065 ± 191; *P* =0.106 ). We considered that the transfection protocol *per se* was neutral in the expression status of CD86. The expression level of CD86 on CD86 siRNA-treated BMDCs was significantly lower than that on non-targeting control siRNA-treated BMDCs [*P* =0.0014] (Figure 1). The expression of CD80 was unaffected by treatment with CD86 siRNA in our preliminary study [9]. These results suggest that treatment with CD86 siRNA specifically suppresses the activation-induced upregulation of CD86 on BMDCs *in vitro*.

### CD86 siRNA treatment impairs BMDCs’ ability to activate Th2 cells

**Figure 2:**
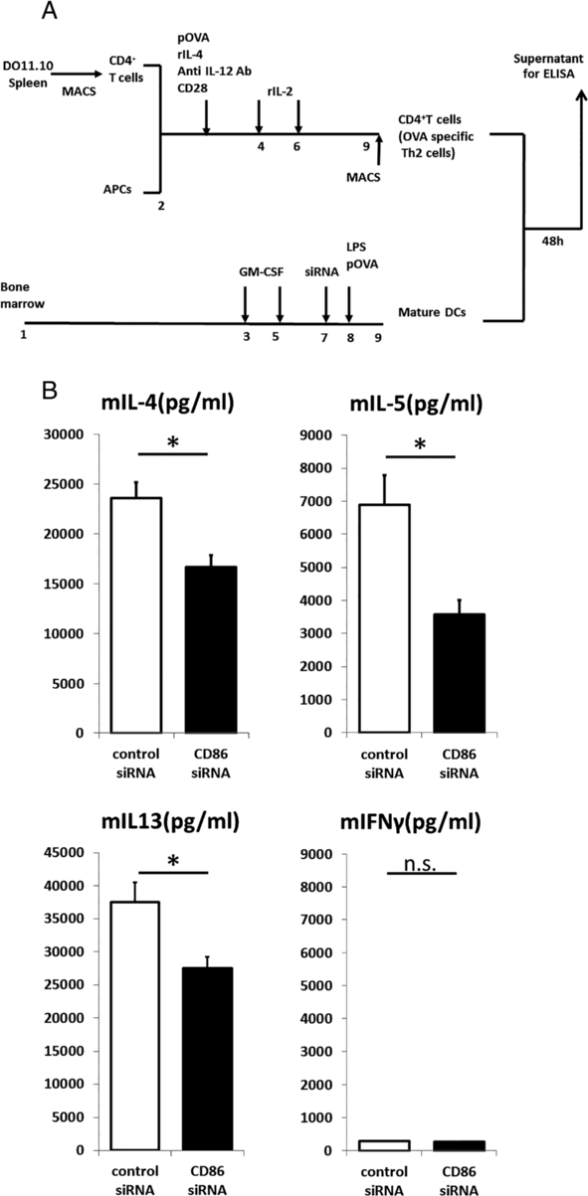
**Th2 cytokine levels in culture supernatant were analyzed by ELISA. (A)** DO11.10 spleen cells were primed with pOVA and APCs in the presence of rIL-4, anti-IL-12 antibody, CD28, and IL-2. Seven days later, CD4-positive cells were purified using MACS and used as effector T cells. CD86 siRNA-transfected BMDCs were pulsed with pOVA and stimulated with LPS and then cultured with effector T cells. **(B)** Coculture supernatants were collected 48 hours later, and cytokine levels were measured by ELISA (n = 4; mean ± SEM). * indicates statistically significant (*P* <0.05) difference between the indicated two groups.

Although many previous studies have shown an essential role of CD86 on APCs in the antigen priming of naïve T cells and subsequent Th2 commitment, there have been no consistent results for the role of CD86 in the reactivation of antigen-specific Th2 cells. OVA-specific Th2 cells were induced by coculture of CD4^+^T cells purified from the spleens of DO11.10 mice with APCs from the spleens of naïve BALB/c mice in the presence of pOVA, recombinant IL-4, neutralizing anti-IL-12 mAb, and agonistic anti-CD28 mAb, and followed by expansion with recombinant IL-2 (Figure 2A). Th2 specificity was confirmed by selective production of IL-4, IL-5, and IL-13, but to a smaller extent, IFN-γ, which is induced by coculture of the cells with pOVA-loaded and LPS-stimulated BMDCs (referred to as pOVA/LPS-BMDCs). The contents of IL-4, IL-5, and IL-13 in the culture supernatants from coculture of the cells with CD86 siRNA-treated pOVA/LPS-BMDCs were significantly lower than those from coculture of the cells with control siRNA-treated pOVA/LPS-BMDCs [IL-4, *P* =0.0209; IL-5, *P* = 0.0209; IL-13, *P* =0.0209] (Figure 2B). When BMDCs were loaded with pOVA but not stimulated with LPS, almost no Th2 cytokine production was detected (data not shown). These results suggest that the reactivation of murine antigen-specific Th2 cells *in vitro* is partially dependent on CD86 on APCs.

### Intratracheal administration of CD86 siRNA downregulates CD86 expression in the airway mucosa

**Figure 3:**
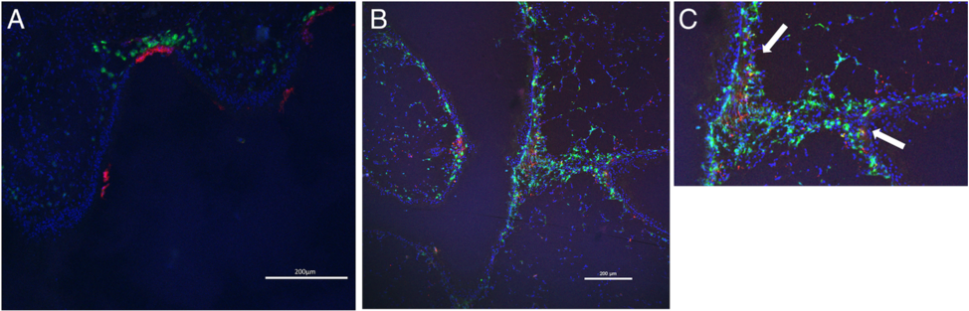
**Distribution of Red-labeled siRNA in the trachea after allergen exposure.** Texas Red-labeled siRNA was administrated intratracheally to sensitized mice. The mice were challenged with OVA 1 hour later. One hour **(A)** or 12 hours **(B)** after allergen exposure, lungs are extracted and stained with DAPI and FITC-conjugated anti-CD11c mAb. Red-labeled siRNA is located in green CD11c-positive cells (arrow).

In an attempt to assess how intratracheally administrated siRNA was distributed to the lung, anesthetized mice were administered with Texas Red-labeled non-coding siRNA and then challenged with OVA. One hour and 12 hours after the challenge, frozen sections were made and stained with FITC-labeled anti-CD11c mAb to detect DCs [15]. One hour after the OVA challenge, Red-labeled siRNA was detected on the airway epithelial layer in a band-like deposition pattern. Green-labeled DCs were clustered around the deposited siRNA (Figure 3A). The immunofluorescence of CD11c^+^cells in proximity of the red siRNA signal was observed in several sites per one section, mainly on the airway epithelial layer. Due to its characteristic distribution, this was unlikely a sectioning/deparaffinizing/staining artifact. Twelve hours after the challenge, the red dots of siRNA were distributed beneath the airway basement membrane and submucosal DCs, indicating that siRNA successfully went through the epithelial barrier (Figure 3B,C). Yellow fluorescence was observed in several sites, suggestive of siRNA taken up by DCs, although a mere topological stratification of fluorescence might not be excluded.

**Figure 4:**
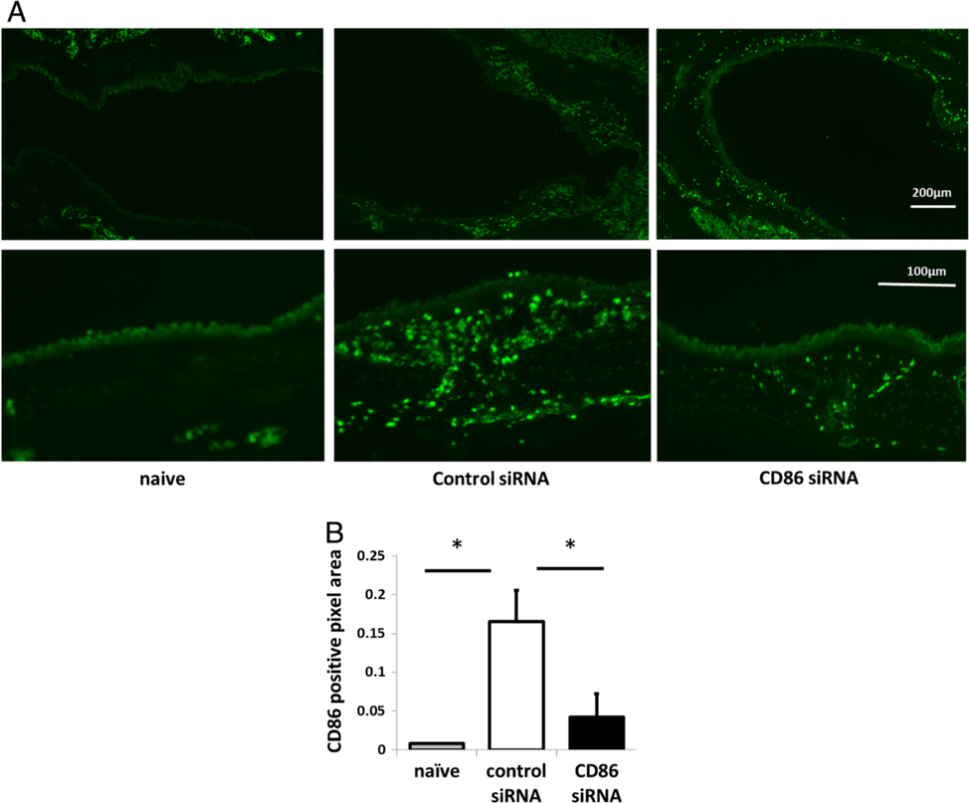
**Effect of siRNA treatment on CD86 positive cells around airway.** Sensitized mice were intratracheally administered with CD86 siRNA or control siRNA and given a single challenge with OVA. Twelve hours after the challenge, the tracheas and lungs were removed and processed for immune-fluorescence study. Naïve mice served as negative control. **(A)** Lung sections from naïve, CD86 siRNA-treated or control siRNA-treated mice stained with FITC-conjugated anti-CD86 mAb. Bar, 200 μm for upper panel; 100 μm for lower panel. The upper panel and lower panel were obtained from different mice **(B)** Micrographs were quantitatively analyzed for the presence of FITC-anti-CD86 signals per unit area with image analysis software. All data mean ± SEM of 3–4 mice per group. * indicates statistically significant (*P* <0.05) difference between the indicated two groups.

Next, for evaluation of the interfering efficacy, sensitized mice were intratracheally administered with CD86 siRNA or control siRNA and given a single challenge with OVA. Twelve hours after the challenge, the tracheas and lungs were removed and processed for immune-fluorescence study. CD86-positive cells were detected by biotin-conjugated anti-CD86 mAb followed by streptavidin-conjugated FITC and assessed semi-quantitatively as CD86-positive areas by image analysis. As shown in Figure 4, in the sections of naïve mice, there were scarce CD86-positive cells in the airway mucosa, although the area of CD86-positive cells was markedly increased in control siRNA-treated and OVA-challenged mice. CD86-expressing cells were detected in dense clusters beneath the airway epithelium. The upregulation of CD86-positive cells was significantly suppressed in the CD86 siRNA-treated mice [*P* =0.0036]. These results demonstrate that local administration of siRNA ameliorates the allergen-induced upregulation of CD86 expression on the airway DCs.

### CD86 siRNA ameliorates allergen-induced asthma phenotypes

**Figure 5:**
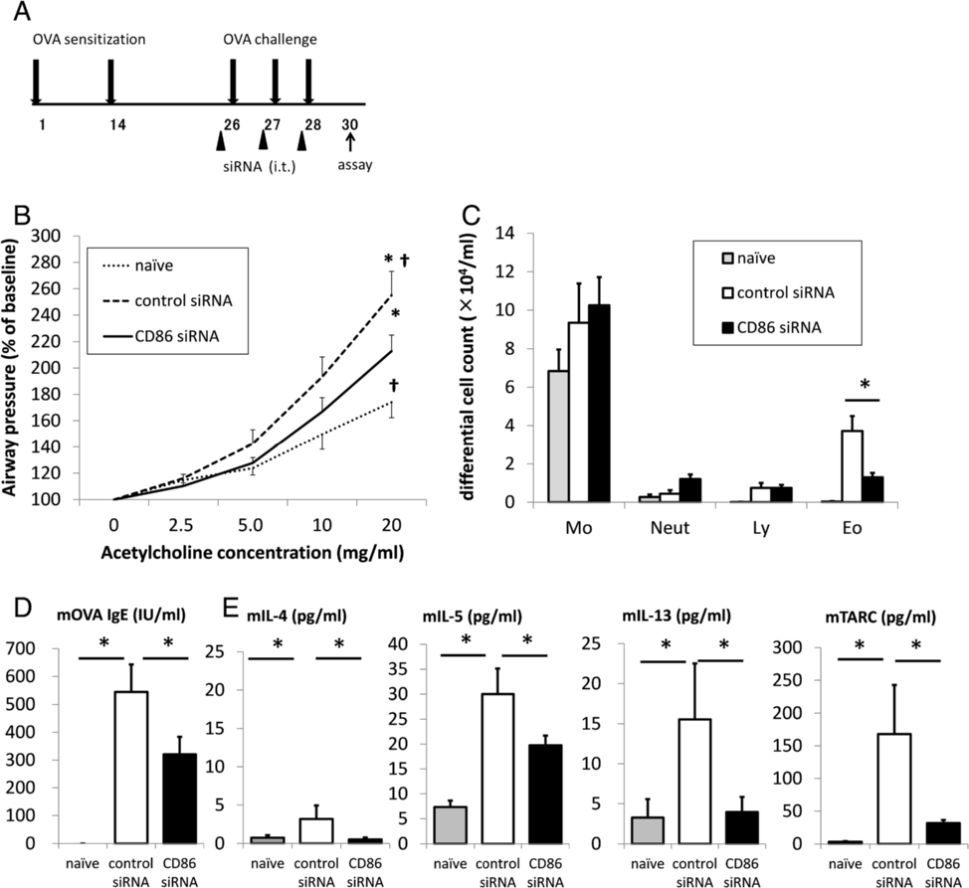
**Effect of treatment with CD86 siRNA on allergic asthmatic response. (A)** CD86 siRNA or control siRNA was administrated intratracheally 1 hour before each OVA challenge. **(B)** Airway hyperresponsiveness to inhaled acetylcholine (ACh) was measured 36 hours after the last OVA challenge. **(C)** Cell counts in BAL fluids were performed. Mo = macrophage; Neut = neutrophil; Ly = lymphocyte; Eo = eosinophil. **(D)** Serum OVA-IgE levels were analyzed. **(E)** Cytokine levels and TARC levels in BAL fluids were analyzed. All data mean ± SEM of 8–12 mice per group. * and † indicate statistically significant (*P* <0.05) difference between the indicated two groups.

**Figure 6:**
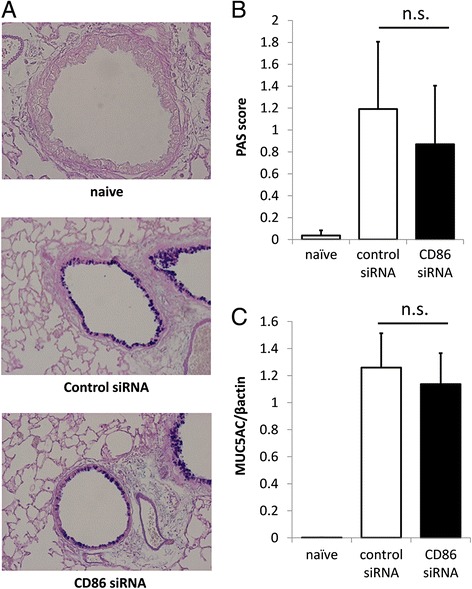
**Lack of effect of treatment with CD86 siRNA on mucus production in OVA-challenged mice. (A)** Lungs were obtained 36 hours after the last OVA challenge and inflated in formalin. Sections were stained with AB/PAS to identify mucus-containing cells. Representative sections from naïve, control siRNA, and CD86 siRNA-treated mice are shown. **(B)** Semi-quantitative analysis of the abundance of PAS-positive cells. The numeric scores for the abundance of PAS-positive, mucus-containing cells in each airway were determined to be as follows: 0, <5% PAS-positive cells; 1, 5–25%; 2, 25–50%; 3, 50–75%; 4, >75%. **(C)** MUC5AC expression in mouse whole lung was analyzed by real-time PCR. The relative levels of the MUC5AC transcripts were presented as fold increase over baseline values. β-actin was taken as a house-keeping gene. All data mean ± SEM of 6–10 mice per group. n.s. means statistically not significant.

To examine the effects of pulmonary CD86 inhibition on asthma phenotypes, CD86 siRNA was administrated one hour before each OVA challenge (Figures 4 and 5). Thirty-six hours after the last OVA challenge, airway hyperresponsiveness to acetylcholine aerosol was measured. Allergen-induced airway hyperresponsiveness in CD86 siRNA-treated mice was significantly lower than that in control siRNA-treated mice [*P* =0.0432]. The number of eosinophils in BAL fluid was significantly lower in CD86 siRNA-treated mice than in control siRNA-treated mice [*P* =0.0117]. The concentrations of IL-5, IL-13, and CCL17 (TARC [thymus- and activation-regulated chemokine], in the old nomenclature) in BAL fluid were significantly lower in CD86 siRNA-treated mice than in control siRNA-treated mice [IL-4, *P* = 0.0101; IL-5, *P* = 0.0339; IL-13, *P* =0.0301; CCL17/TARC, *P* = 0.0479]. The concentration of OVA-specific IgE in the serum was significantly lower in CD86 siRNA-treated mice than in control siRNA-treated mice [*P* =0.0425]. However, the goblet cell hyperplasia and MUC5AC gene expression were not significantly different between CD86 siRNA-treated mice and control siRNA-treated mice (Figure 6). These results suggest that intratracheal administration of CD86 siRNA effectively ameliorates lines of asthma phenotypes, except for goblet cell hyperplasia and mucus hyper-secretion, in a murine model of allergic asthma. In addition, we confirmed that there was no elevation of IL-6 and IFN-β in the serum (data not shown) after CD86 siRNA or control siRNA administration, which eliminated the possibility that control siRNA or CD86 siRNA administration induced nonspecific inflammation.

## Discussion

The present study demonstrated that CD86 siRNA treatment attenuated the upregulation of CD86 on maturing BMDCs. CD86 siRNA-treated BMDCs were poor stimulators of effector Th2 cells, shown by reduced productions of IL-4, IL-5, and IL-13, but not of IFN-γ. *In vivo*, intratracheal administration of CD86 siRNA during OVA challenge reduced the production of IL-5, IL-13, and TARC release and ameliorated airway eosinophilia, airway hyperresponsiveness, and elevation of OVA-specific IgE. These results clearly show that CD86 has a pivotal role in the reactivation of Th2 cells in the effector phase of allergic reactions. In addition, CD86 siRNA treatment did not stimulate systemic production of IL-6 or IFN-β, suggesting that CD86 may become a promising target for the treatment of allergic asthma.

The effector phase of allergic reactions has long been explained by the initiative interaction between antigen-bearing DCs and a memory phenotype of Th2 cells in the draining lymph nodes (DLNs). Thus, airway DCs capture the inhaled allergens, mature with upregulation of costimulatory molecules, including CD86, and then traffic into the DLNs, where they meet with antigen-specific memory Th2 cells. The interaction between those cells leads to clonal expansion and recirculation of effectory Th2 cells, leading to recruitment and activation of effector Th2 cells in the airway mucosa. Although this scenario might be a rationale to interrupt CD86 to CD28 interaction for the treatment of allergic responses, recent investigations have provided a novel concept regarding roles of airway DCs in the activation of Th2 in the airway mucosa. Airway DCs could present antigen directly to memory Th2 cells in the airway mucosa of OVA-sensitized and -challenged mice. Of note, this antigen-presenting activity was accompanied by upregulation of CD86 but not CD80 [16]. Another study demonstrated that allergic lungs specifically retained antigen-bearing DCs within the airway-adjacent region without evidently altering DLN trafficking [17]. This local presentation pathway may be a more feasible target of interruption via intra-airway remedies than is the conventional presentation pathway in the DLNs. A previous study reported that intraperitoneal administration of anti-CD86 mAb during OVA challenge failed to attenuate the subsequent allergic responses in OVA-sensitized mice [18]. The failure might be explained by insufficient distribution of anti-CD86 mAb into the airway mucosa.

Our colleagues have already showed that topical application of cream-emulsified CD86 siRNA ameliorated the clinical manifestations of murine contact hypersensitivity and atopic dermatitis-like disease [9]. It also afford collateral evidence that CD86 has a role in effector phase. But another study reported that intratracheal injection of OVA-pulsed DCs from CD80/CD86 double-deficient mice in OVA-sensitized mice led to the reactivation of Th2 effector responses the same as OVA-pulsed DCs from wild-type mice [19]. siRNA theoretically causes ubiquitous gene silencing, leaving a possibility that CD86 siRNA affects not only airway DCs but also other cell types. CD86 on alveolar macrophages, eosinophils, and B cells was reported to play a role in the development of allergic airway reactions [20,21]. CD86 on B cells stimulates CD28 on T cells and transduces positive signals into B cells that increase IgG_1_ and IgE production. This pathway may be particularly important for memory B cells in which CD86 is upregulated [22,23]. The reduction of OVA-specific IgE in the serum of CD86 siRNA-treated mice in the present study is highly consistent with this pathway.

TARC is the CC chemokine that selectively attracts Th2 lymphocytes toward APCs [24]*.* A previous study showed that TARC expression was highly concentrated in purified lung DCs [25]. In the present study, the mechanism remains unclear as to why the concentration of TARC in BAL fluid was reduced by CD86 siRNA treatment. We confirmed that CD86 siRNA treatment *per se* did not alter the ability to produce TARC in BMDCs *in vitro* (unpublished observation). A possible explanation is that CD86 siRNA treatment reduces recruitment of DCs into the airways by currently unknown mechanism(s), which results in the reduction of TARC in the lungs. The quantitative and qualitative assessments of airway DCs await further investigations.

Contrasting with the effects on lines of asthma phenotypes, CD86 siRNA treatment failed to ameliorate the goblet cell hyperplasia and MUC5AC gene expression, cardinal features of airway remodeling in asthma. A majority of asthma phenotypes, including airway eosinophilia, airway hyperresponsiveness, and airway remodeling, are attributable to the pluripotent effects of IL-13 and its downstream molecules. Those IL-13-mediated phenotypes vary in sensitivity to therapeutic interventions. Airway eosinophilia induced by intratracheal IL-13 was feasibly suppressed by systemic treatment with glucocorticosteroid, while airway hyperresponsiveness and remodeling were resistant to glucocorticosteroid [12]. Similar results were obtained from our recent study that examined the effect of an inhibitor of Janus kinase (JAK), a kinase family mediating multiple cytokine signalings, on OVA-sensitized/challenged mice [14]. In the present study, however, treatment with CD86 siRNA abolished the OVA-challenge-induced elevation of IL-13 in BAL fluid, which makes it difficult to explain the differences in the effects of siRNA via variant sensitivities of IL-13-mediated asthma phenotypes to therapeutic interventions. IL-13-independent mechanisms of airway remodeling must still be elucidated.

To our knowledge, this study is the first report targeting airway DCs for treatment with siRNA. The efficient delivery of siRNA to the target cells has been a challenge for therapeutic application of siRNA since a majority of tissue-constructing cells hardly intake a sufficient amount of siRNA for gene silencing. Given the significant effect of naked siRNA against CD86 in the present study, vigorous phagocytic activity of DCs would be meaningful. Additional carefully designed studies are required to improve pharmacokinetics and facilitate cellular uptake of siRNA [26].

CD86 shares its ligand, CD28, with CD80. In our colleagues’ study, freshly isolated murine CD4^+^T cells were incubated with murine mastocytoma P815 cells transfectants expressing a similar level of either CD80 or CD86 in the presence of anti-CD3 mAb [27]. Both CD80 and CD86 costimulated the proliferation of CD4^+^T cells at comparable time-kinetics and magnitude, but CD86 alone was able to costimulate IL-4 production in CD4^+^T cells. Regarding *in vivo* models, a previous study showed that intranasal administration of anti-CD86 mAb markedly reduced AHR, IgE production, and airway eosinophilia in OVA-sensitized/challenged mice whereas the treatment with anti-CD80 reduced airway eosinophilia alone [28]. On the other hand, another study supported a role for both CD80- and CD86-mediated costimulation in allergen-induced AHR, IgE production, and airway eosinophilia [29]. In the present study, we selected CD86 as a target based on the study indicating that T cell activation during the late-phase airway allergic response is associated with tracheal DC upregulation of CD86 but not CD80 [16]. Concomitant silencing of CD80 and CD86 is subject to future investigation.

The limitation of this animal study around interpretation and translatability to humans may be the choice of OVA as the allergen compared an allergen more relevant to humans, and which has intrinsic activity, such as house dust mite or fungal allergens. Another limitation of this study to the translatability to human asthma may be the question of steroids. Inhaled steroid therapy is used as the mainstay for the treatment of asthma. If steroids effectively down-regulate CD86, the benefit of CD86 siRNA would be minimal. A previous study showed that glucocorticoids inhibited the maturation of human DCs induced by LPS. Thus, glucocorticoids down-regulated the expression of CD86 following LPS stimulation *in vitro* [30]. On the other hand, glucocorticoid insensitivity in some Th2 clones was reversed by blockade of CD86 to CD28 signaling *in vitro* (Dr. Mori A, National Sagamihara Hospital, Japan, unpublished observation). The relevance of CD86-targeted approach in various asthma phenotypes awaits further investigations.

## Conclusion

We have shown that local administration of CD86 siRNA during the effector phase ameliorates lines of asthma phenotypes. Our study also revealed that reduction of TARC level in BALF associated in this mechanism. Targeting airway dendritic cells with siRNA suppresses airway inflammation and hyperresponsiveness in an experimental model of allergic asthma.

## Abbreviations

siRNAs: Short interfering RNAs
OVA: Ovalbumin
BMDCs: Bone marrow-derived dendritic cells
APCs: Antigen-presenting cells
TCR: T cell receptor
DC: Dendritic cell
Ag: Antigens
Tg: Transgenic
LPS: Lipopolysaccharide
AHR: Airway hyperresponsiveness
BAL: Bronchoalveolar lavage
AB/PAS: Alcian blue/periodic acid-Schiff
TARC: Thymus- and activation-regulated chemokine
DLNs: Draining lymph nodes

## References

[1] Kuchroo VK, Das MP, Brown JA, Ranger AM, Zamvil SS, Sobel RA, Weiner HL, Nabavi N, Glimcher LH (1995). B7-1 and B7-2 costimulatory molecules activate differentially the Th1/Th2 developmental pathways: application to autoimmune disease therapy. Cell.

[2] Lenschow DJ, Ho SC, Sattar H, Rhee L, Gray G, Nabavi N, Herold KC, Bluestone JA (1995). Differential effects of anti-B7-1 and anti-B7-2 monoclonal antibody treatment on the development of diabetes in the nonobese diabetic mouse. J Exp Med.

[3] van Rijt LS, Jung S, Kleinjan A, Vos N, Willart M, Duez C, Hoogsteden HC, Lambrecht BN (2005). In vivo depletion of lung CD11c + dendritic cells during allergen challenge abrogates the characteristic features of asthma. J Exp Med.

[4] Vermaelen K, Pauwels R (2003). Accelerated airway dendritic cell maturation, trafficking, and elimination in a mouse model of asthma. Am J Respir Cell Mol Biol.

[5] Gajewska BU, Swirski FK, Alvarez D, Ritz SA, Goncharova S, Cundall M, Snider DP, Coyle AJ, Gutierrez-Ramos JC, Stämpfli MR, Jordana M (2001). Temporal-spatial analysis of the immune response in a murine model of ovalbumin-induced airways inflammation. Am J Respir Cell Mol Biol.

[6] Darcan-Nicolaisen Y, Meinicke H, Fels G, Hegend O, Haberland A, Kuhl A, Loddenkemper C, Witzenrath M, Kube S, Henke W, Hamelmann E (2009). Small interfering RNA against transcription factor STAT6 inhibits allergic airway inflammation and hyperreactivity in mice. J Immunol.

[7] Weckmann M, Collison A, Simpson JL, Kopp MV, Wark PA, Smyth MJ, Yagita H, Matthaei KI, Hansbro N, Whitehead B, Gibson PG, Foster PS, Mattes J (2007). Critical link between TRAIL and CCL20 for the activation of TH2 cells and the expression of allergic airway disease. Nat Med.

[8] Miyamoto S, Hattori N, Senoo T, Onari Y, Iwamoto H, Kanehara M, Ishikawa N, Fujitaka K, Haruta Y, Murai H, Yokoyama A, Kohno N (2011). Intra-airway administration of small interfering RNA targeting plasminogen activator inhibitor-1 attenuates allergic asthma in mice. Am J Physiol Lung Cell Mol Physiol.

[9] Ritprajak P, Hashiguchi M, Azuma M (2008). Topical application of cream-emulsified CD86 siRNA ameliorates allergic skin disease by targeting cutaneous dendritic cells. Mol Ther.

[10] Gu X, Xiang J, Yao Y, Chen Z (2006). Effects of RNA interference on CD80 and CD86 expression in bone marrow-derived murine dendritic cells. Scand J Immunol.

[11] Moriwaki A, Inoue H, Nakano T, Matsunaga Y, Matsuno Y, Matsumoto T, Fukuyama S, Kan-O K, Matsumoto K, Tsuda-Eguchi M, Nagakubo D, Yoshie O, Yoshimura A, Kubo M, Nakanishi Y (2011). T cell treatment with small interfering RNA for suppressor of cytokine signaling 3 modulates allergic airway responses in a murine model of asthma. Am J Respir Cell Mol Biol.

[12] Kibe A, Inoue H, Fukuyama S, Machida K, Matsumoto K, Koto H, Ikegami T, Aizawa H, Hara N (2003). Differential regulation by glucocorticoid of interleukin-13-induced eosinophilia, hyperresponsiveness, and goblet cell hyperplasia in mouse airways. Am J Respir Crit Care Med.

[13] Kawamoto T (2003). Use of a new adhesive film for the preparation of multi-purpose fresh-frozen sections from hard tissues, whole-animals, insects and plants. Arch Histol Cytol.

[14] Matsunaga Y, Inoue H, Fukuyama S, Yoshida H, Moriwaki A, Matsumoto T, Asai Y, Kubo M, Yoshimura A, Nakanishi Y (2011). Effects of a Janus kinase inhibitor, pyridone 6, on airway responses in a murine model of asthma. Biochem Biophys Res Commun.

[15] von Garnier C, Filgueira L, Wikstrom M, Smith M, Thomas JA, Strickland DH, Holt PG, Stumbles PA (2005). Anatomical location determines the distribution and function of dendritic cells and other APCs in the respiratory tract. J Immunol.

[16] Huh JC, Strickland DH, Jahnsen FL, Turner DJ, Thomas JA, Napoli S, Tobagus I, Stumbles PA, Sly PD, Holt PG (2003). Bidirectional interactions between antigen-bearing respiratory tract dendritic cells (DCs) and T cells precede the late phase reaction in experimental asthma: DC activation occurs in the airway mucosa but not in the lung parenchyma. J Exp Med.

[17] Thornton EE, Looney MR, Bose O, Sen D, Sheppard D, Locksley R, Huang X, Krummel MF (2012). Spatiotemporally separated antigen uptake by alveolar dendritic cells and airway presentation to T cells in the lung. J Exp Med.

[18] Haczku A, Takeda K, Redai I, Hamelmann E, Cieslewicz G, Joetham A, Loader J, Lee JJ, Irvin C, Gelfand EW (1999). Anti-CD86 (B7.2) treatment abolishes allergic airway hyperresponsiveness in mice. Am J Respir Crit Care Med.

[19] van Rijt LS, Vos N, Willart M, Kleinjan A, Coyle AJ, Hoogsteden HC, Lambrecht BN (2004). Essential role of dendritic cell CD80/CD86 costimulation in the induction, but not reactivation, of TH2 effector responses in a mouse model of asthma. J Allergy Clin Immunol.

[20] Balbo P, Silvestri M, Rossi GA, Crimi E, Burastero SE (2001). Differential role of CD80 and CD86 on alveolar macrophages in the presentation of allergen to T lymphocytes in asthma. Clin Exp Allergy.

[21] Shi HZ, Xiao CQ, Li CQ, Mo XY, Yang QL, Leng J, Chen YQ (2004). Endobronchial eosinophils preferentially stimulate T helper cell type 2 responses. Allergy.

[22] Podojil JR, Sanders VM (2003). Selective regulation of mature IgG1 transcription by CD86 and beta 2-adrenergic receptor stimulation. J Immunol.

[23] Podojil JR, Kin NW, Sanders VM (2004). CD86 and beta2-adrenergic receptor signaling pathways, respectively, increase Oct-2 and OCA-B Expression and binding to the 3′-IgH enhancer in B cells. J Biol Chem.

[24] Imai T, Nagira M, Takagi S, Kakizaki M, Nishimura M, Wang J, Gray PW, Matsushima K, Yoshie O (1999). Selective recruitment of CCR4-bearing Th2 cells toward antigen-presenting cells by the CC chemokines thymus and activation-regulated chemokine and macrophage-derived chemokine. Int Immunol.

[25] Vermaelen KY, Cataldo D, Tournoy K, Maes T, Dhulst A, Louis R, Foidart JM, Noël A, Pauwels R (2003). Matrix metalloproteinase-9-mediated dendritic cell recruitment into the airways is a critical step in a mouse model of asthma. J Immunol.

[26] Corey DR (2007). Chemical modification: the key to clinical application of RNA interference?. J Clin Invest.

[27] Nakajima A, Watanabe N, Yoshino S, Yagita H, Okumura K, Azuma M (1997). Requirement of CD28-CD86 co-stimulation in the interaction between antigen-primed T helper type 2 and B cells. Int Immunol.

[28] Tsuyuki S, Tsuyuki J, Einsle K, Kopf M, Coyle AJ (1997). Costimulation through B7-2 (CD86) is required for the induction of a lung mucosal T helper (TH2) immune response and altered airway responsiveness. J Exp Med.

[29] Mark DA, Donvan CE, De Sanctis GT, Krinzman SJ, Kbzik L, Linsley PS, Sayegh MH, Lederer J, Perkins DL, Finn PW (1998). Both CD80 and CD86 co-stimulatory molecules regulate allergic pulmonary inflammation. Int Immunol.

[30] Larange A, Antonios D, Pallardy M, Kerdine-Romer S (2012). Glucocorticoids inhibit dendritic cell maturation induced by Toll-like receptor 7 and Toll-like receptor 8. J Leukoc Biol.

